# How does climate change impact health in the African primary care context?

**DOI:** 10.4102/phcfm.v18i1.5474

**Published:** 2026-05-22

**Authors:** Christian Lueme Lokotola

**Affiliations:** 1 Department of Family and Emergency Medicine, Division of Family and Primary Care, Faculty of Medicine and Health Sciences, Stellenbosch University, Cape Town, South Africa; 2 School for Climate Studies, Faculty of Natural Sciences, Stellenbosch University, Cape Town, South Africa

**Keywords:** climate change, primary health care, resilience, mitigation, adaptation, Africa

## Abstract

Primary health care in the African setting is the foundational level for accessing health services; but the quality of services is often challenged by different systemic vulnerabilities. Climate change occurs as a threat multiplier that amplifies these vulnerabilities. This continuing professional development article aims to update the knowledge of primary care providers with evidence-based information on the impact of climate change on health, healthcare services and facilities. The article uses the planetary health framework to explain the pathways from the ecological crisis to its health and social effects. Climate change and pollution are among the global ecological drivers that impact health and society via various proximate causes, such as changes in food production, water quality and quantity, and extreme weather events (e.g. frequent heatwaves, droughts, heavy rainfall and flooding). The effects can be mediated by factors such as wealth, governance, leadership, technology and the strength of the health system. The potential effects on health span the burden of disease from infectious diseases to non-communicable diseases, to mental health problems, maternal and child health, as well as injury and trauma. Social effects such as conflict, displacement, loss of livelihoods and migration have additional effects on health and wellbeing. Primary care providers need to understand how climate change will impact their communities and alter primary care morbidity and mortality. Providers need to prepare, build resilience and explain to patients how climate change is contributing to their health needs and disease patterns.

## Introduction

Climate change has emerged as one of the most pressing determinants of human health in the 21st century. Its impacts mostly occur in regions characterised by environmental vulnerability, socioeconomic inequities and fragile health systems – such as Africa.^1^

**FIGURE 1:**
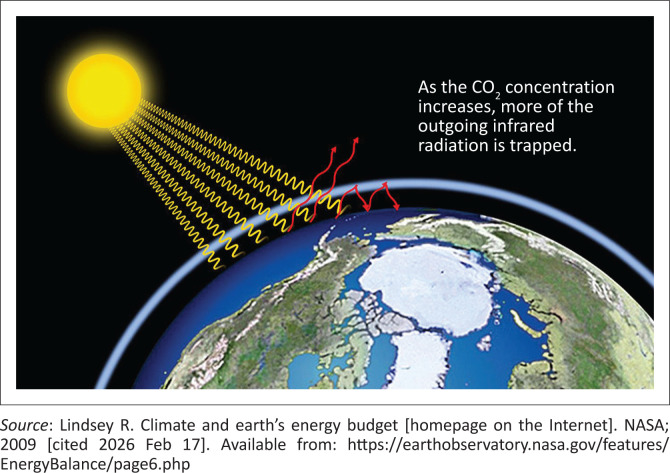
Mechanism for global warming and climate change.

Climate change refers to long-term shifts in climate due to global warming, attributed to an increase in greenhouse gases (GHGs) in the atmosphere.^2^ Climate change is a natural historical phenomenon attributed to solar and orbital variation and can be seen occurring over previous millennia.^3^ However, our current climate change is more dangerous and faster than the natural patterns. The dramatic increase in GHGs is attributed to human activities such as the burning of fossil fuels and industrial-scale agriculture. In healthcare, GHGs also include anaesthetic gases and the propellants in metered dose inhalers. The progressive increase in GHGs, predominantly carbon dioxide, traps the Earth’s reflected infrared solar radiation and heats the atmosphere.^3^ Climate change is driven by several GHGs such as carbon dioxide, methane, nitrous oxide and water vapour which differ in atmospheric lifetime and warming strength (Figure 1).^4^

Global warming, because of increased GHGs, affects the climate in multiple complex ways.^2^ As global temperatures increase, ice sheets and glaciers melt, oceans warm, snow cover decreases and permafrost thaws. Warmer oceans increase evaporation and the atmosphere can contain more water with increased rainfall. There is more energy to fuel hurricanes and cyclones which become more frequent and intense. Ocean currents may be disrupted as the sea warms and disrupt established weather patterns. As more carbon dioxide is captured by the oceans, the oceans also acidify. Sea level rises due to the melting of glaciers and polar ice caps, and the sea also expands when it is warmer. Rising sea level threatens low-lying habitation and causes stronger storm surges. At the same time, rising temperatures can result in more intense heat waves, longer droughts and an increased risk of wildfires.

Communities, therefore, experience climate change as more extreme weather events such as heatwaves, storms or cyclones; and as more subtle changes in seasons, temperatures and rainfall. These changes can also promote environmental hazards such as flooding or wildfires.^1^ For example, the recent drought in the Horn of Africa,^5^ the annual cyclones impacting Mozambique and Malawi,^6^ the changed seasonal migration patterns of pastoralists in Nigeria can all be partly attributed to underlying climate change.^7^

## How the ecological crisis leads to health and social effects

**FIGURE 2:**
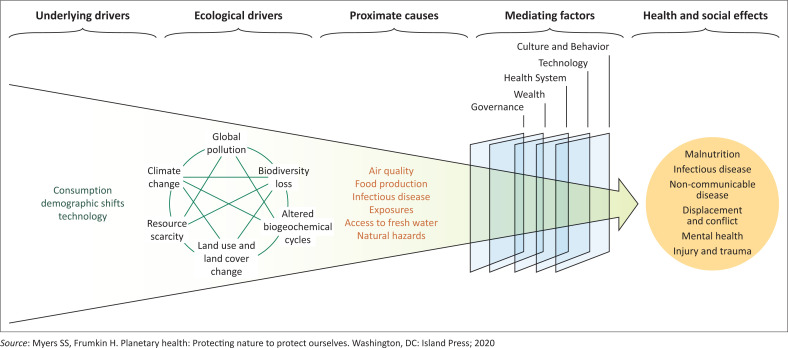
The planetary health framework.

These changes have adverse impacts on both human and animal health, and the One Health movement acknowledges these inter-relationships. Most recently, the coronavirus disease 2019 (COVID-19) pandemic was characterised as a zoonotic infection and global pandemic, illustrating the dangers when viruses jump from animal to human populations.^8^ There has also been a growing interest in planetary health that focuses on the health and social effects of our ecological crisis as shown in Figure 2.^9^ We will use this framework to elaborate on the pathway from climate change to its health and social effects.

### Underlying drivers

The ecological crisis has been driven over the last century by the rapid rise of industrialisation and excessive consumption of limited planetary resources, globalisation and its rapid diffusion of intensive carbon products, as well as the rise in global population. For example, the global population has risen from 2 billion in 1925 to 8 billion in 2023 and is expected to reach 10 billion this century.^10^ Population growth drives consumption and production of GHGs, although there is a decrease in the rate of growth that suggests we will reach a peak later this century.^11^

### Ecological drivers

Climate change is not the only ecological hazard impacting the health of our communities but is one of several interrelated drivers. For example, the use of fossil fuels has also driven the advent of plastics and led to widespread concern about global plastic pollution and the potential adverse effects of microplastics on our health.^12^ There has been a massive loss of biodiversity, and the collapse of fisheries has led to a new global agreement to protect 30% of our oceans so that life can recover.^13^ Land has been given over to monoculture with deforestation, soil erosion and altered biogeochemical cycles from massive use of chemical fertilisers.

### Proximate causes

The ecological crisis does not directly impact health but does so through several proximate causes. For example, many cities experience poor air quality with particulate pollution that impacts respiratory and cardiovascular diseases. Higher temperatures are associated with more allergens in the air.^14^ Food production is impacted by damage from weather events, drought, changes in pests and diseases, and even changes in the nutritional value of foods. Changes in weather impact the habitats of infectious disease vectors, such as mosquitoes, with an overall increase in communities at risk.

Change in temperature, precipitation patterns and extreme weather events directly affect the contamination of water sources, the efficacy of sanitation systems and the viability of hygiene behaviours.^15^ Increased temperatures trigger extreme rainfall events and floods which lead to loss and damage of infrastructure whilst driving poor water quality and lack of sanitation. Droughts can also reduce the amount of water available that then becomes polluted, also leading to an increase in water-borne diseases.^16^ Natural hazards themselves can have direct effects on people and their health services.

### Mediating factors

Although climate change is global, countries and communities are not all equal in terms of their vulnerabilities and capacities to withstand the proximate causes.

In Africa, vulnerability arises from the interaction of environmental exposure and socioeconomic fragility. African countries are mostly low or middle-income countries with weaker infrastructure and limited resources. Africa’s vulnerability also stems from high dependence on climate-sensitive livelihoods (e.g. subsistence farming, pastoralism, informal sector jobs)^17^ and under-resourced health systems, particularly primary health care. The poorest and most marginalised groups, such as people living in informal settlements, migrants, rural smallholders, women, children and the elderly, face disproportionate exposure. Vulnerability is structurally embedded in poverty, infrastructure deficits and limited adaptive capacity.^18^ The region’s exposure to recurrent droughts, flooding, temperature variability and shifting rainfall patterns intersects with high burdens of infectious disease, widespread poverty, rapid population growth and climate-sensitive livelihoods.^16^ This creates a high baseline burden of infectious diseases coupled with emerging non-communicable diseases.

### The health and related social effects of climate change

#### Vector-borne diseases (e.g. malaria, dengue, others)

Several reviews recommend urgent integration of climate information into vector control planning. Increased temperatures may increase transmission of vector-borne diseases in Africa, mostly in highland or fringe areas.^19^ Rising temperatures under climate scenarios affect vector ecology through temperature, rainfall and humidity. This contributes to shifting the distribution, seasonality and intensity of vector-induced diseases such as malaria and dengue. Several reviews and modelling studies indicate complex and location-specific impacts mostly in the tropical regions, whilst drought or extreme rainfall can either reduce or amplify risk depending on context and vector species.

#### Water-, sanitation- and hygiene-related diseases

Waterborne and hygiene-sensitive diseases constitute a major portion of the climate change burden in Africa. The Intergovernmental Panel on Climate Change (IPCC) and regional reviews link extreme hydrometeorological events with diarrhoeal disease burdens across Africa.^1^ Many communities rely on unimproved water sources. Although there may be various modifying factors, such as individual level of education, the impact of extreme weather events on water can amplify the risk of diseases such as gastroenteritis, cholera, typhoid fever and other enteric pathogens, mostly in urban informal settlements lacking piped water and sewage systems.^20^

#### Food security and malnutrition

Studies show that climate change through droughts, floods and shifting rainfall has severe impacts on crop yields, livelihoods, food safety and security, driving acute and chronic malnutrition.^21^ Food security refers to all stages of the food chain, production, processing, distribution and consumption to prevent health risk in food, whilst food safety refers to access to sufficient and nutritious food to meet people’s dietary needs and preferences. Both terms encompass availability, accessibility and stability of crop yields and food to combat mal- and undernutrition. Drought exacerbated by El Nino has produced large-scale food insecurity and child malnutrition in Africa, illustrating climate-driven poor health outcomes.^21^

#### Non-communicable diseases

Traditionally, health systems in Africa have focused on communicable diseases such as malaria, human immunodeficiency virus (HIV) and/or acquired immunodeficiency syndrome (AIDS) and tuberculosis. With climate change and air pollution playing a significant role in disease occurrence, Africa may undergo an epidemiological transition with a rapid rise of non-communicable diseases.^14^ The IPCC and Lancet Countdown emphasise the combined effects of heat and air pollution on morbidity and mortality.^2^ African urbanisation and reliance on biomass and coal energy have increased levels of coarse particulate matter (PM_10_) and fine (PM_2.5_) particulates in several countries. Dust storms worsen urban air pollution and are exacerbated by climate-related heat and drought.^22^ A study conducted in Cape Town, South Africa, demonstrated the interaction effect of climate-related extreme temperatures on air pollution and vice versa on respiratory and cardiovascular diseases.^23^ Heat stress increases heart rate, blood viscosity and risk of heart attacks and strokes, even insulin resistance and diabetes onset, especially in urban areas with high pollution.

Significant threats to maternal health and pregnancy outcomes are under recognised in most African countries. Pregnant women are especially vulnerable to impaired placental blood flow that contributes to adverse outcomes like pre-term birth and low birth weight, preeclampsia and hypertensive disorders.^24^ During pregnancy, heat stress, drought and dehydration can increase metabolic and cardiovascular demand that can trigger early labour and impair foetal growth.^25^

#### Mental health and climate change impacts

Throughout the entire African continent, climate shocks such as storms, droughts and extreme heat drive psychological distress. Due to social and economic constraints, the population experiences breakdowns in social support, as well as loss and damage to their livelihoods. Although the field of climate change and mental problems is not fully explored in Africa, the direct effects are thought to be related to immediate trauma and injury, and the indirect effects are through damage to the social foundation of society.^26^

A recent review of the mental health effects on adolescents and young adults found that extreme weather events can be associated with depression, acute stress, post-traumatic stress disorder, substance use, suicide risk and anxiety disorders. Emotional responses to climate change include sadness, suicidal ideation, ecological grief and loss of one’s identity.^27^

#### Migration, vulnerability and climate change impacts

Migration refers to internally displaced people (IDP), migrants (people moving from one country to another), refugees (legally recognised migrants in the country of host) and asylum seekers (not yet legally recognised as a refugee in the country of destination).^28^ Their movements are not only driven by socioeconomic push and pull factors. Environmental factors such as extreme weather events (droughts, floods, cyclones, heatwaves) alter their livelihoods or force people to seek a better life elsewhere.^29^ But they often remain vulnerable when their movements are accompanied with overcrowding, inadequate water, sanitation and hygiene.^30^ Poor-quality water supplies in camps or informal settlements lead to rapid spread of water-borne diseases such as cholera or diarrhoea. Overcrowded and poor shelters can easily trigger respiratory diseases such as asthma and chronic obstructive pulmonary disease (COPD). Most migrants and IDP experience significant but under-recognised and under-assessed mental health issues such as anxiety, depression and PTSD.^31^ They face physical violence from the host country, which often underlines tension over job competition, housing and services between migrants and local people. Women and children are the most affected in these events, with limited antenatal care and inadequate nutrition.^31^ All these health risks are often aggravated when migrants and IDP are denied access to healthcare services (barriers to medication and interruption of chronic care, for diabetes, hypertension and HIV) in the host country.^30^

#### Conflict, injury and trauma

Several studies state that climate change is increasingly recognised not only as an environmental and health threat, but also as a risk multiplier that can escalate social tensions, contribute to violent conflict and increase injury and trauma. When governance is weak, climate change can increase competition over scarce water and grazing land and lead to economic instability and loss of livelihoods with migration and local tensions.^32^ Populations may compete for grazing land and water with conflict between farming and pastoralist communities. Extreme weather events can directly or indirectly cause injury, trauma, domestic violence, interpersonal aggression and death.^33^ Climate change-induced wildfires are directly associated with burns and smoke inhalation. Floods can lead directly to drowning and physical injury. Heatwaves can contribute directly to heatstroke and cardiovascular injury.^3^ Drought can trigger loss and damage of livestock, food insecurity and increased recruitment into violent groups, particularly in pastoralist regions.^34^

#### Health effects of vulnerable health facilities and services

Increased vulnerability of healthcare facilities and services has compromised the ability of the health system to respond to communities’ health emergencies. In most National Adaptation Plans for climate change risks, the health sector and its infrastructure are considered a vulnerable sector.^35^ The healthcare sector and services are facing increased stress related to the intensified impacts of climate change on health infrastructure. For example, Mozambique recently reported that 472 health facilities had been destroyed since 2019.^36^ Extreme weather events can destroy hospitals and clinics, prevent healthcare workers from getting to work and damage medical equipment. This results in reducing, disrupting and delaying emergency response capacity. After disasters from floods, cyclones, heatwaves, wildfires or drought, increased morbidity and mortality often result from interruption of healthcare services.^37^ This is particularly visible at the primary care level which has become not only highly sensitive to climate shocks, but also the frontline of defence against the health impacts of climate change.^38^ Primary care facilities are the foundation of health systems, especially in low- and middle-income countries. When infrastructure is damaged or inaccessible due to climate shocks, routine and continued healthcare stops or is disrupted.^37^ This can generate long-term health consequences beyond the immediate disaster, and contribute to increased workload and burnout for healthcare workers.

## Conclusion

While there is no new or specific climate disease, climate change will impact the whole burden of disease. Climate change is part of a broader ecological crisis that impacts health through a variety of proximate causes. The impact of these proximate causes is modulated by various contextual factors. Many African communities are vulnerable and will be significantly affected by climate change-related health and social effects. Climate change is a threat multiplier for infectious diseases, non-communicable diseases, malnutrition, mental health, injury and trauma, maternal and child health problems, as well as migration and conflict. Changes in morbidity and mortality will be experienced at the primary care level, which is the entry portal to the entire health system. Therefore, all primary care providers, including family physicians, need to be aware of how climate change will impact their practices. Thus, primary care can serve as a critical point for adaptation, mitigation and community resilience against climate change and its health effects. Strengthening the resilience of healthcare infrastructure is therefore essential to protect health gains and ensure the continuous delivery of essential services in the face of climate change.
